# First use of silicon carbide detectors with graphene-enhanced contacts for medical dosimetry

**DOI:** 10.1038/s41598-024-56544-x

**Published:** 2024-03-13

**Authors:** Ivan Lopez Paz, Celeste Fleta, Faustino Gomez, Diego Miguel González, Giulio Pellegrini

**Affiliations:** 1 Instituto de Microelectronica de Barcelona, IMB-CNM-CSIC, Cerdanyola del Valles, 08193 Barcelona, Spain; 2 Departamento de Física de Partículas, Universidade de Santiago de Compostela, 15782, Santiago de Compostela, Spain

**Keywords:** Characterization and analytical techniques, Electrical and electronic engineering, Electronic and spintronic devices

## Abstract

Silicon Carbide (SiC) is a radiation hard wide bandgap semiconductor, which makes it an interesting alternative for radiation detector fabrication, with potential applications such as High Energy Physics, synchrotron and radiotherapy instrumentation. In addition, by reducing the amount of metal over the active area of said detectors (typically used for electrical connectivity with the implant of the pn-junction) unwanted effects from secondary interactions which can affect the accuracy of the measurement can be diminished, essential to meet the medical standards of precision. In this article, the use of epitaxially-grown graphene is explored as an alternative to metallic contacts with these prototypes. To this end, the first prototypes of SiC diodes with epitaxial graphene contacts were produced at IMB-CNM for radiation detection,along with reference devices. In order to characterise the feasibility of the technology in the medical application, the dose rate linearity of the SiC device with graphene was measured in a radiotherapy Linac in the dose rate range of 1–6 Gy/min. The response of the device was compared to that observed on devices with similar geometries reported elsewhere. To fully characterise the devices, the same exercise was repeated in a laboratory X-ray tube. Under the later set-up, the prototype is compared against a device with a fully metallised active region.

## Introduction

The interest of the use of Silicon Carbide (SiC) for the fabrication of radiation detectors has been growing in the past few years with thanks to the increasing availability of high quality 4-8 inch epitaxial SiC wafers. Such material showcases interesting properties such as a high thermal conductivity and higher displacement energy threshold than Silicon - which makes it effectively a more radiation hard material.

Such properties make Silicon Carbide an attractive material to fabricate radiation detectors e.g. for High Energy Physics ^1,2^ as well as neutron detection applications ^3^, were they need to sustain high levels and long periods of irradiation. In addition, low dark currents and insensitivity to visible light due to its wide band-gap energy and its better tissue equivalence with respect to silicon make a good candidate for dosimetry in medical environments. In particular, there is a recent growing interest in Ultra High Dose Rate (UHDR) irradiation, a promising new radiotherapy approach to achieve the FLASH effect ^4–6^ which has been demonstrated to reduce toxicity in healthy tissue while keeping the same tumour control probability ^4^. To achieve this effect, pulses of 100 kGy/s to 10 MGy/s in less than 200 ms are delivered, therefore requiring fast and radiation hard instrumentation, with excellent accuracy. Note that in this work, only conventional radiotherapy conditions are explored as a proof-of-principle.

Typically, diodes are fabricated with metallic layers across their active region in order to obtain an ohmic contact with the electrode implant. For the performance standards of medical dosimetry, the presence of metal is detrimental, since it can introduce multiple-scattering, therefore affecting the device response. A possible alternative to utilising metallic contacts is to replace that layer with graphene ^7^ - an electrically conductive mono-atomic carbon layer.

The implementation of a graphene layer for its use in radiation Silicon ^8^ sensors has been previously explored, e.g. deep ultraviolet photodetection ^9,10^, to reduce the absorption of the impinging light before reaching the sensitive volume. Similarly, graphene layer implementation in Silicon Carbide by means of epitaxial growth has been studied ^11^, which opens the possibility of fabricating metal-less radiation detection. Other approaches are being studied in parallel using carbon-based contacts to achieve metal-less dosimeters, in which diamond is used as the semiconductor material and graphite (typically introduced by laser graphitisation ^12^) as the conductive contact for medical dosimetry ^13^. However, albeit diamond outperforms SiC in the thermal conductivity and radiation hardness in addition to being tissue-equivalent, SiC is more cost effective and available in larger areas.

In this paper, the use of graphene as a conductive alternative to achieve the contact is explored for medical dosimetry Quality Control. To this end, SiC detectors with epitaxially-grown graphene layer substituting the metallic contact over the active region produced at IMB-CNM are studied as the first prototypes with this geometry. This technology has been studied in the context of charged particle and UV light field detection ^14^, showing promising results.

## Silicon carbide detector samples

Aluminum-doped Silicon Carbide diodes were produced in a quarter of a SiC wafer with a 50 $\mu$m epitaxial layer to test the technology. A graphene layer was grown in the p^+^-implant side in a high temperature process, allowing for the sublimation of the Silicon atoms and the rearrangement of the Carbon atoms left ^11,15^.

**Figure 1:**
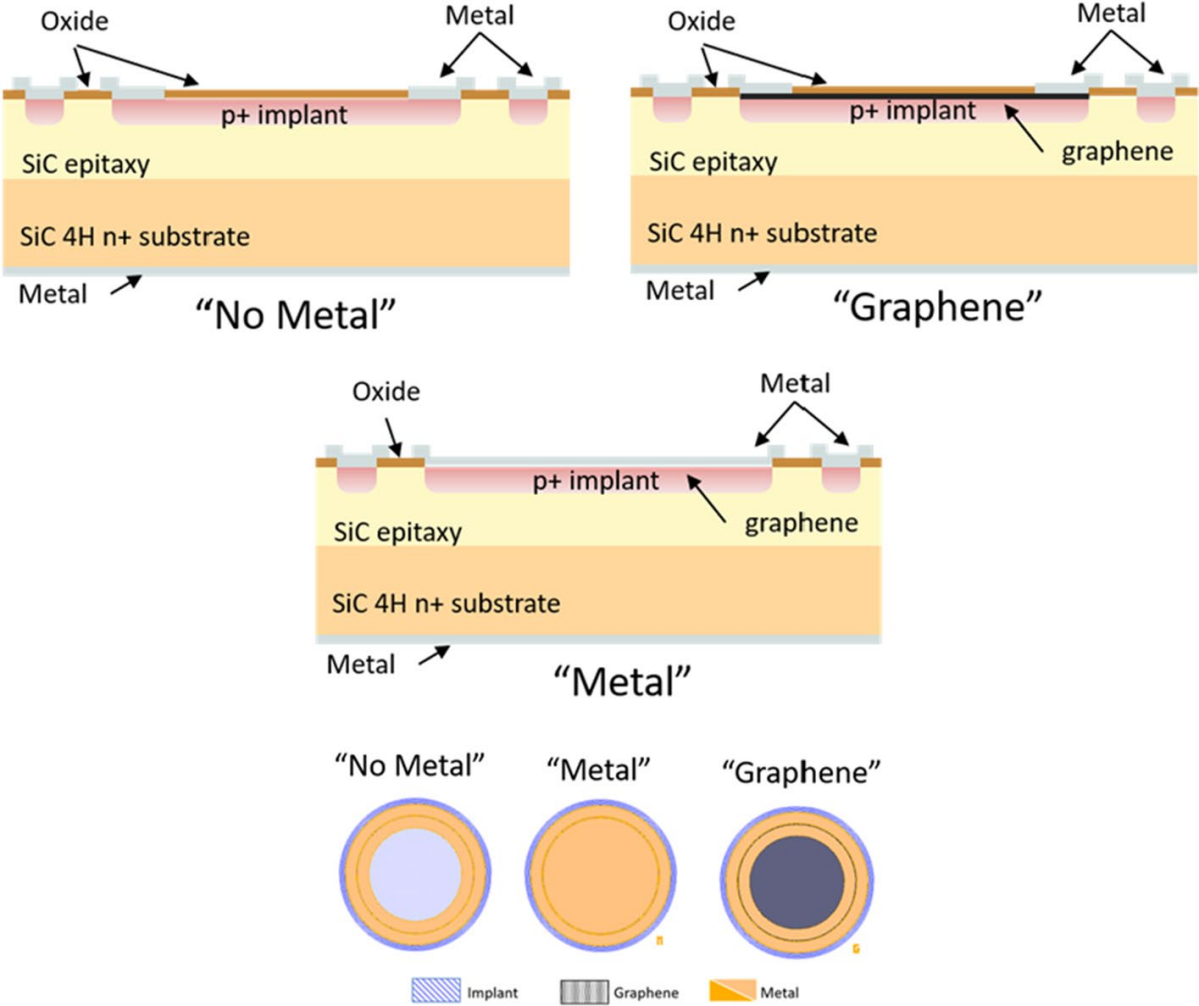
Cross-section (top) and front structure schematics (bottom) of the three SiC detector sample designs.

Among other structures, one-millimeter diameter diodes were fabricated in the wafer, featuring metallic contacts with the pn-junction (hereafter “Metal” sample), a 800 $\mu$m diameter window (“No Metal”) and containing a layer of epitaxial graphene (EG) for the contact with the junction (“Graphene”) (the presence of which was confirmed via Raman spectroscopy), buried in a metallic ring for electrical connectivity of the sensor via wirebonding. The metallic components, including the collection rings from “No Metal” and “Graphene” samples, in the front surface are a stack of Ti/Pt/Au (20 nm/100 nm/100 nm), based on former investigations ^16^, while the backside is fully covered with Ti/Ni/Au (50 nm/15 nm/50 nm). Figure 1 shows a sketch of the cross-section for each of the samples. A sketch of the cross-section and top-view of the three samples is shown in Fig. 1.

**Figure 2:**
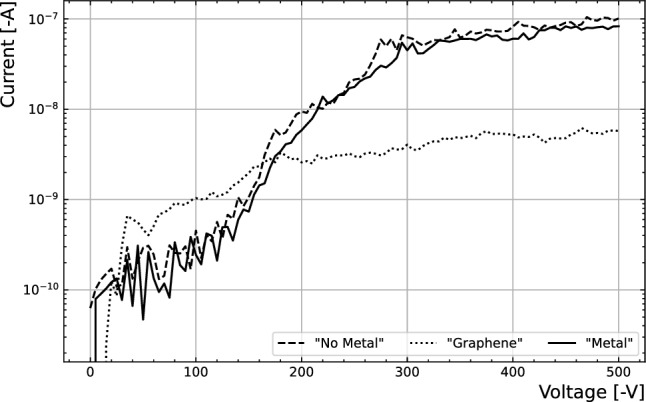
Current as a function of bias voltage of the three samples considered in this paper.

These samples were glued onto ceramic PCBs by means of a conductive silver glue. To determine the functionality of the devices after packaging, the current-voltage characteristics of the three devices were measured, as shown in Fig. 2. All three devices showcased currents below the sensitive range of the measuring device (Keithley 2410 Source Meter Unit) at 0 V - the operational voltage in the following experiments.

The sensors and their respective wirebonds were covered with a $\sim$1 mm layer of quick set transparent epoxy adhesive in some cases. Notice that this packaging and metal choice is not optimal for radiotherapy due to the high-Z materials on them, in particular the metal stack over the active area of the “Metal” sample and the bias rings, which can contribute to the reading, in particular at low energies, via secondary interactions with the X-rays.

In addition to the SiC devices, a commercial PTW Semiflex ionisation chamber is used for calibration purposes. A summary of the detectors used for the studies presented in this paper can be found in Table 1.

**Table 1:** Collection of samples utilised in the experiments in this paper.

Device	Active area surface	Packaging	Linac (6 MV)	X-ray chamber	Comment
“No Metal”	Al-doped SiC	Ceramic PCB	Output factor	Dose rate linearity	Req. pre-irradiation at Linac ($\sim$60 Gy) and X-ray tube.
					Permanent instability after epoxy.
“Graphene”	Graphene over Al-doped SiC	Ceramic PCB Epoxy-covered	Dose rate linearity	Dose rate linearity	Stable after epoxy and $\sim$40 Gy pre-irradiation. Similar response to “No Metal” at Linac
“Metal”	Ti/Pt/Au metal stack over Al-doped SiC	Ceramic PCB Epoxy-covered	Not measured	Dose rate linearity	Stable, 2.3$\times$ the response of “Graphene” at X-ray tube
Ionisation chamber	Graphite walls	PMMA covered standard form-factor	Ref. output factor	–	Commercial, PTW Semiflex Ion Chamber

## Methods

**Figure 3:**
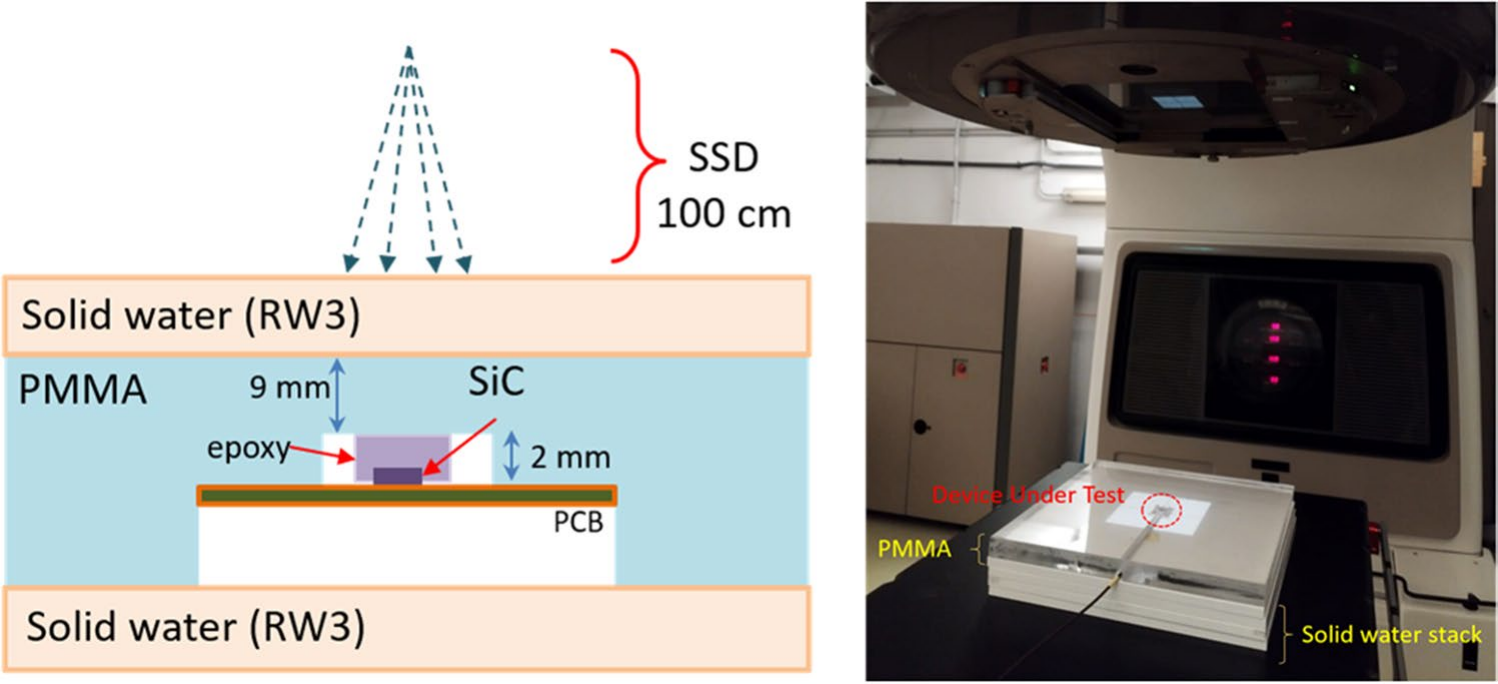
Schematic of the set-up (left) and a picture of the device within the PMMA slab mounted below the source of the Linac (right). Note that the top solid water slabs in the set-up were not pictured for illustration.

In order to study the suitability of EG-SiC diodes for radiotherapy Quality Assurance, the “Graphene” prototype was tested in a radiotherapy Linac. In this set-up, the beam energy was fixed to 6 MV, with a dose rate in the range of 1–6 Gy/min, and a fixed irradiation field of 10$\times$10 cm^2^. The sample was mounted inside a 21 mm-thick PMMA (polymethyl methacrylate) plate, where 9 mm of this material was on top of the device. A stack of 35 mm of solid water (RW3) slabs were placed directly on top of the PMMA. This geometry was chosen to achieve a controlled 5 g/cm^2^ mass-density equivalent to water, to which the set-up dose rate was calibrated. The source-surface distance (SSD) was fixed to 100 cm. A drawing of the set-up is shown in Fig. 3.

The output factor is defined as the dose observed by the target relative to the one observed with a reference field (10$\times$10 cm^2^), which is sensitive to the difference in the dose from scattering due to the Linac collimator aperture ^17^. Under the same set-up shown in Fig. 3, with the “No Metal” sample, the output factor was measured by irradiating the device with a fixed dose rate (6 Gy/min) and energy (6 MV), varying the irradiation field. The charge response at different field sizes, from 5$\times$5 to 30$\times$30 cm^2^ are normalised to the response obtained with a field of 10$\times$10 cm^2^ and compared to that of a reference detector–a commercial PTW Semiflex ionisation chamber.As the active area of the device was not covered by epoxy, the solid water stack was corrected for by removing a 1 mm slab to recover the same 5 g/cm^2^ mass-density equivalent to water, matching the reference detector data. The goal of this measurement is to demonstrate the benefits of removing the metal layer in the energy response.

To study the the response and linearity to the beam current samples were tested with an X-ray tube (Hamamatsu L9421-02). They were irradiated with an operating voltage of 60 kV and beam currents in the range of 60–120 $\mu$A. To do this, the samples were installed on the movable stage available within the X-ray tube cabin, utilising the aforementioned PMMA slab, at 10 cm from the source. A 1.3 mm Aluminum plate was placed over the PMMA slab surface to filter low energy radiation from the source.

A summary of the experimental set-ups and the devices used in each of them can be found in Table 1.

In all cases the devices were read out with a portable Sun Nuclear PC electrometer with an operational bias voltage of 0 V. Detector response was measured for each condition by integrating the charge collected at 30 second intervals, before the start of the irradiation ($Q_{before}$), at least three 30 s charge measurements with the beam on ($Q_{beam}$), and repeating the background measurement immediately after the beam is turned off ($Q_{after}$). Each set of measurements is then corrected for background contributions by removing the average of the two background measurements - i.e. $Q_{corr}=Q_{beam}-\frac{1}{2}(Q_{before}+Q_{after})$.

To determine the dose rate and beam current linearity, a straight line function was fitted to the charge as a function of dose rate data, and the residuals (i.e. the difference between the data and the fitted function) were calculated to measure the deviation from linearity. An alternative linearity test with the same data is performed by adjusting a linear function with an exponent as a free parameter: $Q=K\cdot D^{\Delta }+Q_{dark}$, ^18^ where *D* is the dose rate or beam current.

## Results

The “Graphene” sample was installed under the Linac beam to test its dose rate linearity. Due to the non-optimal packaging of the device, and to avoid surface currents distorting the measurement, the sample was fully covered by a 1 mm thick layer of quick set epoxy adhesive. During the curing process, a slow decay in leakage current of the device was observed, which plateaued after 4 h from the deposition of the epoxy.

**Figure 4:**
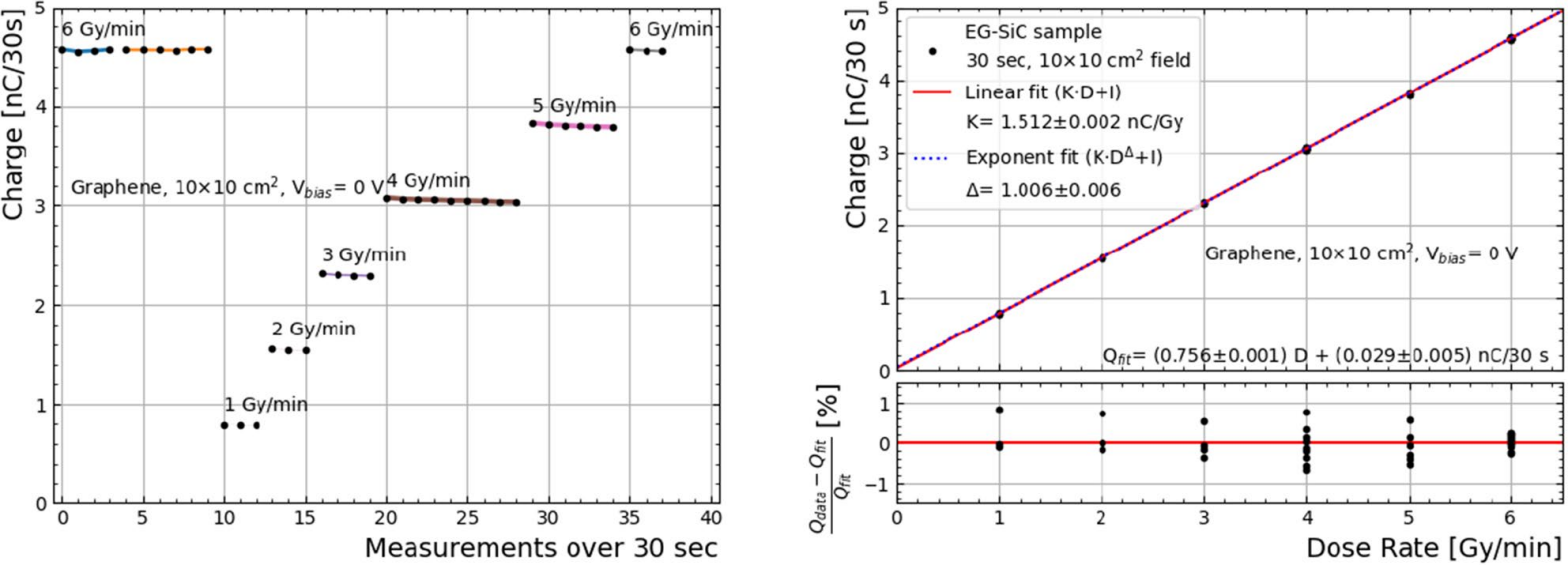
Charge measurement sequence (left) and integrated charge over 30 seconds (right) obtained with the epoxy-covered “Graphene” sample with a $V_{bias}$=0 V, with a 10$\times$10 cm^2^ field and 6 MV energy. A linear and an exponential fit of the data are shown in red and blue, respectively. The deviation from linearity is shown below for each measurement. A dose linearity of better than 1% is observed.

The sample was then irradiated at different dose rates, with a fixed field of 10$\times$10 cm^2^ and beam energy of 6 MV. At the start of the measurements the device had accumulated over 40 Gy from earlier tests. The integrated charge over each 30 second measurement are shown in Fig. 4(left), showing a good repeatability.

Figure 4(right) shows these measurements as a function of dose rate. All measurements deviate less than 1% from the straight line fit (which falls within the medical standards) across the full range of dose rates in standard radiotherapy. The slope of the function is found to be 1.512±0.002 nC/Gy at a bias voltage of 0 V, which is compatible with measurements performed with 3 $\mu$m epitaxy SiC 1-mm diameter diodes with 20 MeV electrons ^19^. As a result of the exponent function curve adjustment, the exponent parameter is found to be $\Delta$=1.006±0.006, and therefore confirming that the response is linear within the uncertainties.

**Figure 5:**
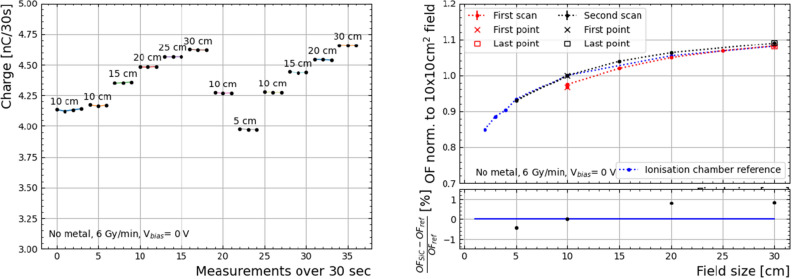
Charge measurement sequence (left) and integrated charge over 30 seconds (right) obtained with the epoxy-free “No Metal” sample with a $V_{bias}$=0 V normalised to a field of 10$\times$10 cm^2^. The data was taken with a dose rate of 6 Gy/min and 6 MV energy at different fields in the Linac, and is split in two scans, at measurement number 18. The output factor of a reference ionisation chamber detector is overlapped. An agreement of within percent-level is achieved when considering the second half of data points, when the behaviour of the “No Metal” device is seemingly stable.

The output factor was measured with the “No Metal” device utilising the Linac as described in the previous section, i.e. normalising the response at different field sizes to the response obtained with a 10$\times$10 cm^2^ field. The measurements were split in two data sets (see Fig. 5), since the device showed indications of requiring pre-irradiation of about 60 Gy to achieve repeatability in the measurements. The second data set is taken as reference. As a result, when comparing the normalised response to the results from a reference ionisation chamber, the agreement of the output factor is at the percent level. Notice that the charge response at the reference field (in Fig. 5(left)) is consistent to that of the “Graphene” sample (Fig. 4).

**Figure 6:**
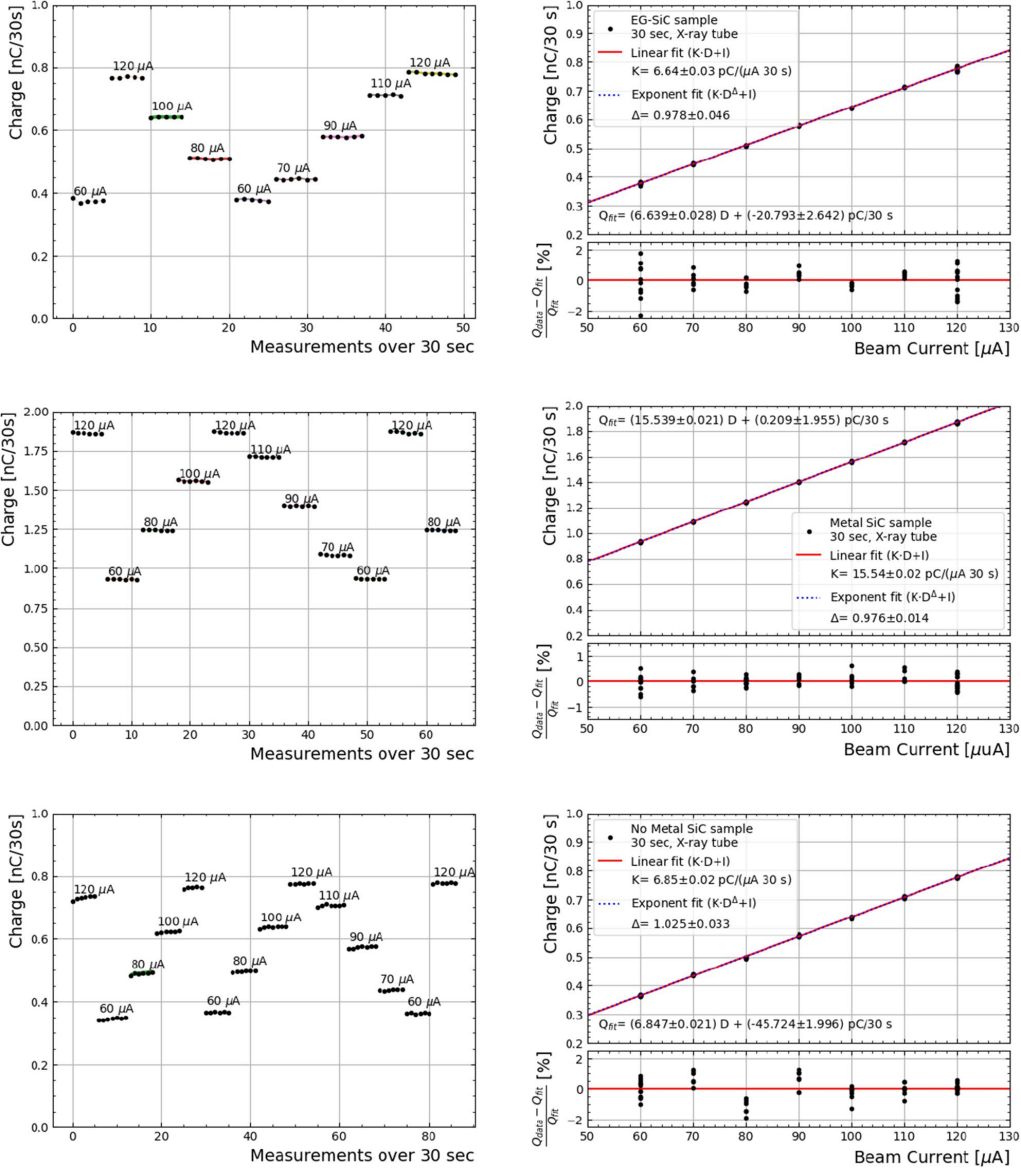
Charge measurement sequence (left) and integrated charge over 30 seconds (right) obtained with the “Graphene” (top), “Metal” (middle) and “No Metal” (bottom) samples with a $V_{bias}$=0 V, under an X-ray tube with a voltage of 60 kV. A linear and an exponential fit of the data are shown in red and blue, respectively. The deviation from linearity is shown below for each measurement. “No metal” sample was only measured without epoxy, and only data after measurement 30 were considered for the fit.

The dose linearity experiment was repeated with the devices installed in the X-ray tube as described in the previous section. The linearity and response was characterised over a beam current range of 60 to 120 $\mu$A, with a source voltage of 60 kV. The results of which, are to be compared with the performance of the other samples (“No Metal” and “Metal”) in the same conditions. The resulting beam current response is shown in Fig. 6, with their corresponding fit results. Only results obtained with “No Metal” sample before epoxy deposition are reported, since the stability of its response degraded afterwards. We observed a stable behaviour of the “Metal” sample without the need of pre-irradiation.

**Figure 7:**
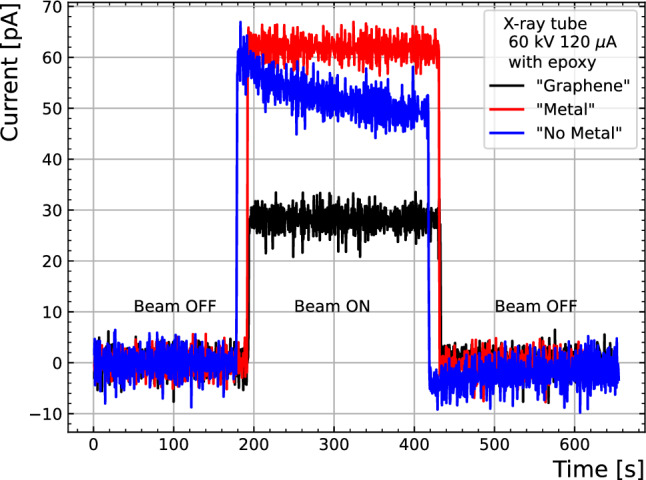
Current measured under an X-ray tube with 60 kV and 120 $\mu$A for the three devices covered in epoxy. The response of the “No Metal” sample is attributed to the not well cured epoxy.

All samples show a percent-level accuracy, although the “No Metal” sample required $\sim$30 measurements to stabilise its response and therefore, all measurements previous to that were excluded for its linearity calculation. Similar behaviour was observed in data taken in the Linac facility, as aforementioned. Moreover,the “No Metal” sample suffered from persistent unstable behaviour after being covered by epoxy, as can be observed in Fig. 7, where the current is shown for the three devices while irradiating for 4 min after background subtraction. There, the “No Metal” sample showcases an overshoot increase of the current which decays over the course of the irradiation. This is attributed to a not well cured epoxy resin, and has been observed by the authors in similar devices with different radiation fields.

In all cases, the current fluctuation is at the level of 2–2.5 pA, which corresponds to 10% of the signal of the “Graphene” sample and 4% of the “Metal” sample. A higher signal to noise ratio in the “Metal” device could explain a better performance in terms of linearity compared to the obtained results with the “Graphene” sample under the X-ray tube (see Fig. 6).

**Figure 8:**
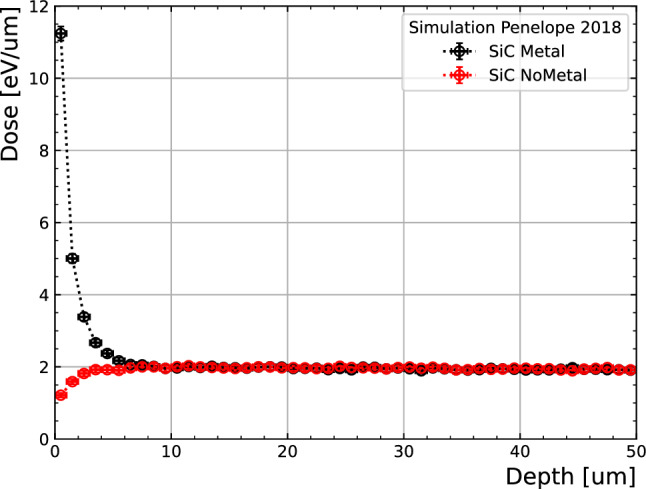
Dose deposited on the first 50 $\mu$m Silicon Carbide with and without the presence of the metal stack, as simulated in PENELOPE ^20^.

Indeed, the response observed by the “Metal” sample is noticeably larger (2.3 times) than its no-metal counterparts under the same conditions. This effect could be attributed to the presence of high-Z metallic layers over the active region, artificially increasing the dose in the first micrometers of the sensor. This is confirmed with a Monte Carlo simulation by means of the PENELOPE software ^20^. The dose deposition with a source from a 60 kV X-ray tube on a Silicon Carbide slab after a 1.2 mm Al, 10 mm of PMMA and, when applicable, a metal stack was simulated. To this end, the energy distribution of the photons after the 150 $\mu$m Beryllium window featured in the X-ray tube was obtained with the XCOMP5R software ^21^. The mono-atomic layer of graphene was not included as it has negligible effect for the purpose of the simulation. The dose as a function of depth on the first 50 $\mu$m of SiC are shown in Fig. 8 for a sample with and without the metallic stack. The simulation shows an increased dose exposure in the first micrometers after the metallic stack, effectively modifying the irradiation field in the active volume, thus leading to higher currents. This is caused by the higher interaction probability for low energy photons of the set-up (<60 keV) from the X-ray tube on the high-Z metallic layers, causing low energy secondary radiation which is absorbed in the SiC. It is important to note that, when operating the sensors with a bias of 0 V, the depletion depth due to the built-in voltage in the pn-junction is expected to be lower than 10 $\mu$m ^22^, therefore the relative difference in dose between the “Metal” and “No Metal” configurations is higher as the devices are sensitive only in the first micrometers from the implant–thus explaining the discrepancy in response across the devices.

## Discussion

The work presented in this paper shows the first characterisation of graphene-enhanced Silicon Carbide diodes for use in the medical setting.

The “Graphene” sample showcases an excellent linearity response when utilised in a medical standard radiotherapy Linac, within the 1 % level. In the X-ray tube set-up, it is also able to perform with percent-level accuracy. In the latter setting, the “Metal” sample performs at a comparable level than that of the “Graphene” sample, with the drawback of an artificial increase of its response (about 2.3 times), which is attributed to the presence of high-Z metals covering the full active region. Admittedly, an optimised selection of materials for the contact metal stack could reduce this effect. In addition, the “No Metal” sample shows a percent-level agreement with a reference ionisation detector in the output factor within the range of 5 to 30 cm fields, although this device suffered from instabilities.

Although future work is required to finalise the characterisation of the technology and optimisation of the detector design (e.g. metal stack choice) and packaging (e.g. PCB design) are possible, these first results show that SiC devices with graphene contacts are a suitable alternative in medical dosimetry.

## Data Availability

The data that support the findings of this study available from the corresponding author on reasonable request.
